# Harpagide Confers Protection Against Acute Lung Injury Through Multi-Omics Dissection of Immune–Microenvironmental Crosstalk and Convergent Therapeutic Mechanisms

**DOI:** 10.3390/ph18101494

**Published:** 2025-10-04

**Authors:** Hong Wang, Jicheng Yang, Yusheng Zhang, Jie Wang, Shaoqi Song, Longhui Gao, Mei Liu, Zhiliang Chen, Xianyu Li

**Affiliations:** 1 Beijing Key Laboratory of Traditional Chinese Medicine Basic Research on Prevention and Treatment for Major Diseases, Experimental Research Center, China Academy of Chinese Medical Sciences, Beijing 100700, China; wanghong0569@163.com (H.W.); yjc18143609206@163.com (J.Y.); yushengzhang271727@foxmail.com (Y.Z.); wang_jie921@163.com (J.W.); lm1692276021@163.com (M.L.); 2 School of Basic Medicine, Shanxi University of Chinese Medicine, Jinzhong 030619, China; m18053676639@163.com; 3 Fujian Provincial Key Laboratory of Pien Tze Huang Natural Medicine Research and Development, Zhangzhou Pien Tze Huang Pharmaceutical Co., Ltd., Zhangzhou 363000, China; pzhglh@zzpzh.com

**Keywords:** acute lung injury, acute respiratory distress syndrome, Harpagide, single-cell transcriptomic, multi-omics

## Abstract

**Background:** Acute lung injury (ALI) and its severe form, acute respiratory distress syndrome (ARDS), remain major causes of morbidity and mortality, yet no targeted pharmacological therapy is available. Excessive neutrophil and macrophage infiltration drives reactive oxygen species (ROS) production and cytokine release, leading to alveolar–capillary barrier disruption and fatal respiratory failure. **Methods:** We applied an integrative multi-omics strategy combining single-cell transcriptomics, peripheral blood proteomics, and lung tissue proteomics in a lipopolysaccharide (LPS, 10 mg/kg)-induced mouse ALI model to identify key signaling pathways. Harpagide, an iridoid glycoside identified from our natural compound screen, was evaluated in vivo (40 and 80 mg/kg) and in vitro (0.1–1 mg/mL). Histopathology, oxidative stress markers (SOD, GSH, and MDA), cytokine levels (IL-6 and IL-1β), and signaling proteins (HIF-1α, p-PI3K, p-AKT, Nrf2, and HO-1) were quantitatively assessed. Direct target engagement was probed using surface plasmon resonance (SPR), the cellular thermal shift assay (CETSA), and 100 ns molecular dynamics (MD) simulations. **Results:** Multi-omics profiling revealed robust activation of HIF-1, PI3K/AKT, and glutathione-metabolism pathways following the LPS challenge, with HIF-1α, VEGFA, and AKT as core regulators. Harpagide treatment significantly reduced lung injury scores by ~45% (*p* < 0.01), collagen deposition by ~50%, and ROS accumulation by >60% relative to LPS (*n* = 6). The pro-inflammatory cytokines IL-6 and IL-1β were reduced by 55–70% at the protein level (*p* < 0.01). Harpagide dose-dependently suppressed HIF-1α and p-AKT expression while enhancing Nrf2 and HO-1 levels (*p* < 0.05). SPR confirmed direct binding of Harpagide to HIF-1α (KD = 8.73 µM), and the CETSA demonstrated enhanced thermal stability of HIF-1α. MD simulations revealed a stable binding conformation within the inhibitory/C-TAD region after 50 ns. **Conclusions:** This study reveals convergent immune–microenvironmental regulatory mechanisms across cellular and tissue levels in ALI and demonstrates the protective effects of Harpagide through multi-pathway modulation. These findings offer new insights into the pathogenesis of ALI and support the development of “one-drug, multilayer co-regulation” strategies for systemic inflammatory diseases.

## 1. Introduction

ALI is a common and life-threatening clinical syndrome characterized by a high incidence and mortality rate. Its pathogenesis is complex, involving excessive inflammatory responses, oxidative stress imbalance, and programmed cell death. A multicenter study in the United States reported a 28-day mortality rate as high as 41% among 2466 patients with moderate to severe ALI; during the early phase of the COVID-19 pandemic, the mortality rate among ICU patients with ARDS reached 67–85%, and during the 2002–2003 SARS outbreak, it was approximately 52% [1].

Currently, treatment for ALI and ARDS primarily relies on supportive care, including lung-protective ventilation strategies such as low tidal volume ventilation, appropriate positive end-expiratory pressure, prone positioning, conservative fluid management, and, when indicated, the use of neuromuscular blocking agents [2,3,4].

Despite extensive clinical investigations, no pharmacological agent has yet demonstrated a definitive capacity to reduce ALI/ARDS-related mortality or improve long-term outcomes. A major contributing factor is that the majority of ALI cases originate from an uncontrolled cytokine storm, which frequently escalates into systemic inflammatory syndrome [5].

The LPS-induced murine model is widely employed to recapitulate the inflammatory microenvironment characteristic of ALI and ARDS. LPS, a major component of the outer membrane of Gram-negative bacteria, activates Toll-like receptor 4 (TLR4) and initiates downstream signaling cascades including NF-κB, MAPK, and JAK/STAT pathways. This signaling leads to a robust release of pro-inflammatory cytokines (e.g., TNF-α, IL-1β, IL-6) and chemokines (e.g., CCL2/MCP-1, CXCL1/KC, CXCL2/MIP-2, CXCL8/IL-8), which establish spatial gradients within the pulmonary microenvironment. These gradients provide directional cues for circulating neutrophils and monocytes, which sense them via chemokine receptors, undergo cytoskeletal reorganization and polarization, and subsequently migrate across the endothelium toward areas of highest chemokine concentration. The resulting accumulation of leukocytes in lung tissue drives the inflammatory cascade and contributes to the pathological features of ALI [6,7,8].

Due to its dense capillary network, large surface area, and direct exposure to the external environment, the lung is particularly susceptible during cytokine storms and often becomes the first and most severely affected organ. Activated neutrophils and macrophages recruited to the pulmonary compartment release large amounts of ROS and proteolytic enzymes. These mediators disrupt the integrity of the alveolar-capillary barrier, increase vascular permeability, and promote the development of pulmonary edema and extensive inflammatory infiltration [9]. Collectively, these pathological processes contribute to the onset of ALI and its progression to ARDS. The excessive activation of immune cells and consequent ROS accumulation further amplify oxidative stress. Oxidative stress not only causes direct structural damage to cellular membranes, proteins, and nucleic acids but also activates pivotal stress response pathways, including the KEAP1-NRF2 axis, p38 MAPK, and PI3K/AKT signaling networks, thereby intensifying cellular stress responses and influencing cell fate decisions [10,11,12,13].

Accordingly, elucidating the common regulatory mechanisms governing immune cell migration, pulmonary immune infiltration, and lung microenvironmental interactions under cytokine storm conditions is critical for the development of therapeutic strategies capable of targeting multiple immune cell types, signaling pathways, and pathological processes simultaneously. Integrative dissection of multi-dimensional immune regulatory networks may facilitate the identification of interventions that achieve “one drug, multilayer co-regulation” effects, offering substantial theoretical and translational value for improving clinical outcomes in ALI and associated systemic complications [14].

Based on the preliminary screening and multi-omics integrative analyses conducted by our research group, Harpagide was identified as a candidate monomer compound targeting the shared key regulatory mechanisms. Harpagide is an iridoid glycoside predominantly found in species of the Scrophulariaceae and Lamiaceae families, including *Harpagophytum procumbens* DC. ex Meisn. (devil’s claw), *Verbascum* L., *Scrophularia ningpoensis* Hemsl., and other *Scrophularia* species. These plants have been historically employed in traditional medicine for their anti-inflammatory, antioxidative, and antirheumatic properties [15]. Extracts from *Harpagophytum procumbens* DC. ex Meisn. exhibit potent antioxidant activity in vitro, attenuating Fe^2+^- and nitroprusside-induced lipid peroxidation and restoring endogenous antioxidant defenses [16]. Recent studies have demonstrated that Harpagide suppresses Angiotensin II-induced microglial activation via inhibition of the TLR4/MyD88/NF-κB signaling pathway, thereby reducing neuronal apoptosis and improving blood–brain barrier integrity [17]. Moreover, its structurally related compound, harpagoside, exerts anti-inflammatory effects through downregulation of COX-1/2 and iNOS expression, leading to a reduction in pro-inflammatory mediators [18].

To systematically elucidate the shared key regulatory mechanisms underlying immune cell migration, pulmonary immune cell infiltration, and interactions with the lung microenvironment driven by the cytokine storm, and to address the current gap in this field, we conducted an integrated multi-omics analysis. This study combined whole-blood proteomics, lung tissue proteomics, and single-cell transcriptomics of pulmonary immune cells, with a specific focus on macrophage and neutrophil subsets. Through multi-level and multi-dimensional analyses, we comprehensively characterized the coordinated actions of these immune cells in the pathological process, identified common regulatory mechanisms across different cell types and tissue compartments, and validated the findings through both in vivo and in vitro models.

## 2. Results

### 2.1. Proteomic Profiling of Lung Tissues and Harpagide-Induced Alterations in LPS-Induced ALI

**Figure 1:**
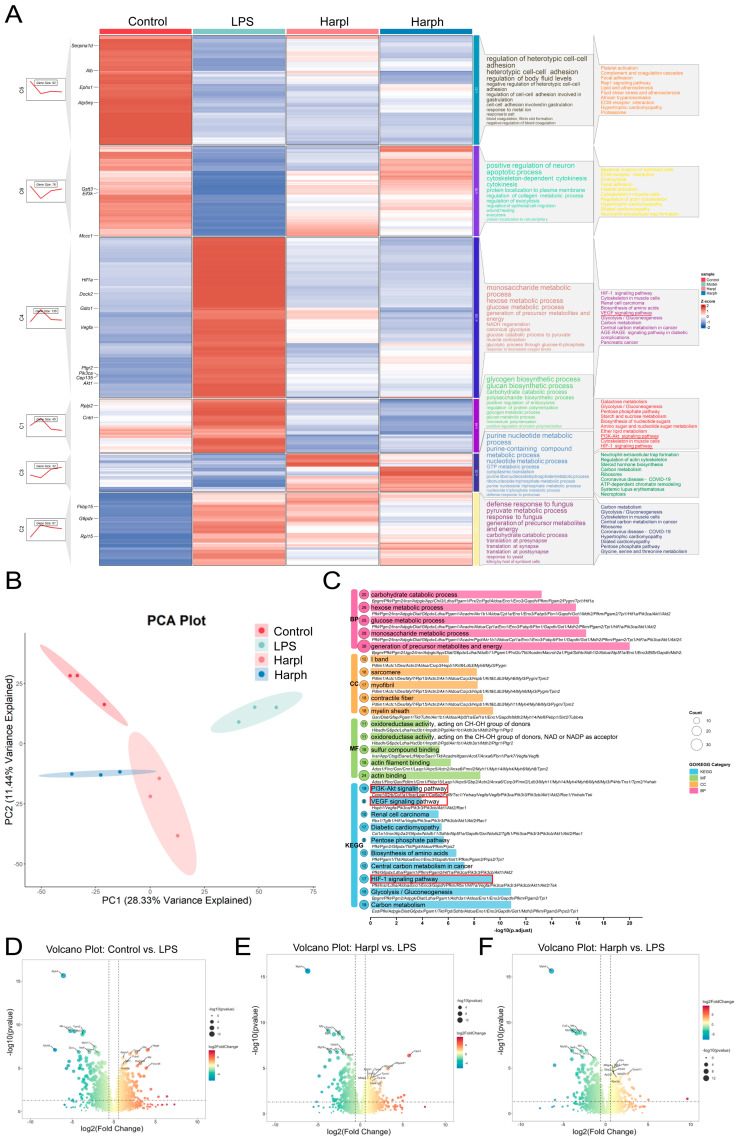
Proteomic profiling of lung tissues and Harpagide-induced alterations in LPS-induced ALI. (**A**) Trend heatmap of differentially expressed proteins generated, with key pathways highlighted by red lines. (**B**) PCA plot showing distinct separation among the Control group, the LPS group, and the groups treated with Harpagide. (**C**) Enrichment map summarizing KEGG and GO pathway analyses based on trend-consistent proteins, with key pathways highlighted by red boxes. (**D**–**F**) Volcano plots showing differentially expressed proteins in the comparisons of LPS vs. Control (**D**), HarpL vs. LPS (**E**), and HarpH vs. LPS (**F**), with dotted lines indicating the thresholds of *p* < 0.05 and |log_2_FC| > 0.585.

PCA demonstrated clear separation among the Control, LPS, and Harpagide-treated groups (HarpL and HarpH) in proteomic expression profiles (Figure 1B). Control, LPS, and Harpagide-treated groups (HarpL and HarpH) formed distinct clusters, supporting significant differences in proteomic expression profiles under different conditions. Notably, the Harpagide-treated groups showed higher correlation with the Control group than with the LPS group, suggesting a potential restorative effect of Harpagide on LPS-induced proteomic alterations. Volcano plots (Figure 1D–F) revealed substantial changes in protein expression between the LPS group and other treatment groups, highlighting the top ten upregulated and downregulated proteins. Several proteins involved in inflammation and oxidative stress, including Thbs1, Mtf1, and Gsta4, were significantly dysregulated in the LPS group. Notably, Harpagide treatment at both HarpL and HarpH doses partially reversed the expression of these key proteins. Among them, Opa1, Gbp4, Gsta4, and Vim have been well documented to play critical roles in ALI and oxidative stress responses. These findings suggest that Harpagide may exert its protective effects by modulating inflammation- and stress-related signaling pathways [19,20,21,22,23,24].

Trend analysis of differentially expressed genes was conducted using the ClusterGVisR package. Among the identified gene clusters, clusters 1, 2, 3, 4, and 6 displayed expression patterns that were consistent with disease progression and therapeutic response. Specifically, genes in these clusters were significantly upregulated or downregulated in the LPS group compared with the control group, and these alterations were reversed in a dose-dependent manner following Harpagide treatment. Only cluster 5 did not follow this pattern. Genes from the trend-consistent clusters were selected for subsequent Kyoto Encyclopedia of Genes and Genomes (KEGG) and Gene Ontology (GO) enrichment analyses (Figure 1A).

KEGG analysis revealed significant enrichment of the VEGF, HIF-1, and PI3K–Akt signalling pathways, all of which were upregulated in the LPS group and downregulated after treatment. Core regulators, including HIF-1α, VEGFA, PIK3CA and AKT, followed this expression trend. GO enrichment analysis of the same gene set showed that in the Biological Process (BP) category, enriched terms were primarily associated with carbohydrate metabolic processes. In the Cellular Component (CC) category, enrichment was observed in components related to muscle structure and contractile fibres. In the Molecular Function (MF) category, significantly enriched terms included oxidoreductase activity, particularly those acting on CH-OH groups of donors, and sulfur compound binding, indicating a potential involvement of oxidative stress (Figure 1C).

### 2.2. Proteomic Profiling of Whole Blood and Harpagide-Induced Alterations in LPS-Induced ALI

**Figure 2:**
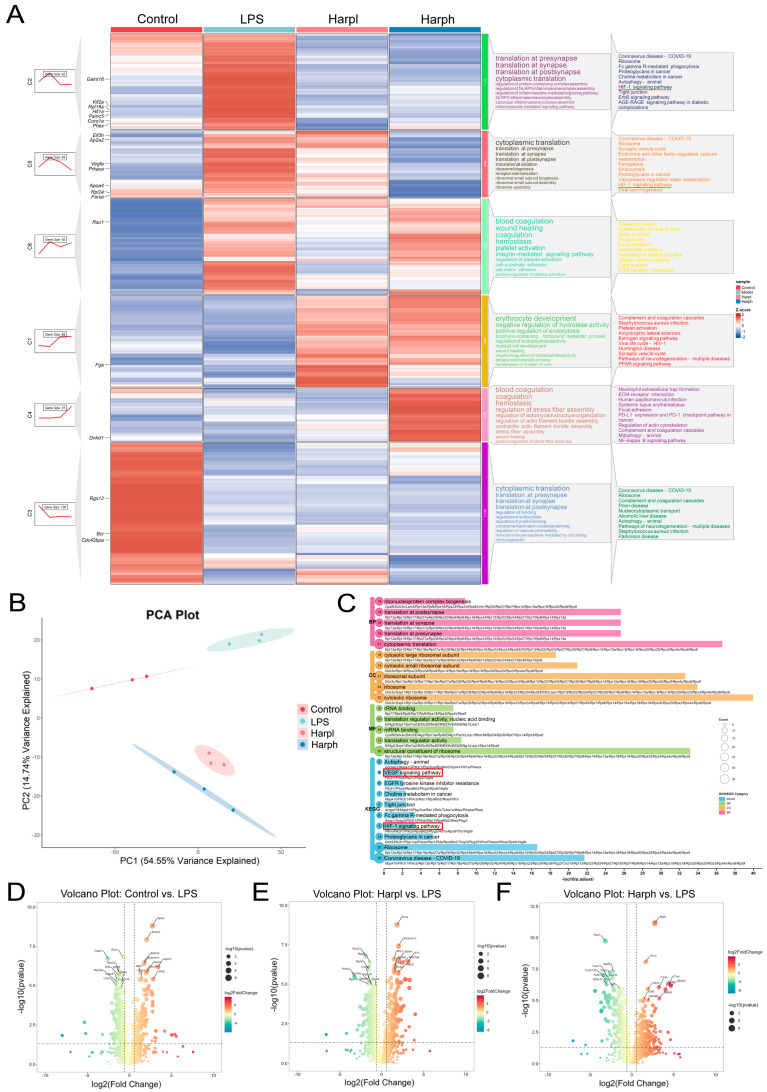
Proteomic profiling of whole blood and Harpagide-induced alterations in LPS-induced ALI. (**A**) Trend heatmap of differentially expressed proteins, with key pathways highlighted by red lines. (**B**) PCA plot showing distinct separation among the Control group, the LPS group, and the groups treated with Harpagide. (**C**) Enrichment map summarizing KEGG and GO pathway analyses based on trend-consistent proteins, with key pathways highlighted by red boxes. (**D**–**F**) Volcano plots showing differentially expressed proteins in the comparisons of LPS vs. Control (**D**), HarpL vs. LPS (**E**), and HarpH vs. LPS (**F**), with dotted lines indicating the thresholds of *p* < 0.05 and |log_2_FC| > 0.585.

PCA of Whole blood proteomics revealed clear separation among the Control, LPS, and Harpagide-treated groups (HarpL and HarpH). The LPS group deviated markedly from the Control group along the PC1 axis, indicating significant LPS-induced proteomic alterations. Both Harpagide-treated groups formed distinct clusters, with the HarpH group showing a closer shift toward the Control group compared to HarpL, suggesting a dose-dependent restorative trend (Figure 2B). Volcano plots illustrate the distribution of differentially expressed proteins in whole blood among groups. Compared with the Control group, the LPS group displayed significant upregulation of several proteins related to inflammation and immune activation, including Apoe, C1qa, C1qb, Bpifa2, and C1qc (D). Upon Harpagide treatment, both HarpL (E) and HarpH (F) groups exhibited modulation of these proteins. Notably, proteins such as Pura and Prdx2 known to be involved in oxidative stress and immune responses [25,26], were differentially expressed following Harpagide administration, indicating a potential anti-inflammatory and antioxidant effect (Figure 2D–F).

Trend analysis of differentially expressed genes was performed using the ClusterGVisR package. Among the identified clusters, clusters 2 and 5 showed expression patterns that aligned with disease progression and therapeutic response. Genes in these clusters were significantly upregulated or downregulated in the LPS group compared with the control group, and these changes were reversed in a dose-dependent manner following Harpagide treatment. These genes were subsequently used for KEGG and GO enrichment analysis (Figure 2A).

The KEGG analysis again highlighted significant enrichment in the VEGF, and HIF-1 signalling pathways, all of which were upregulated in the LPS group and downregulated after treatment. Key regulators such as HIF-1α and VEGFA followed the same trend, consistent with the proteomic results from lung tissue. GO enrichment analysis of the same gene set revealed strong enrichment in ribosome-related and translation-associated biological functions (Figure 2C).

Given that immune cell mobilisation, particularly of macrophages and neutrophils, is a key feature of the cytokine storm triggered by LPS in whole blood, we next examined the underlying mechanisms at single-cell resolution.

### 2.3. Single-Cell Transcriptomic Analysis Reveals Neutrophil Heterogeneity and Inflammation-Associated Changes in ALI

**Figure 3:**
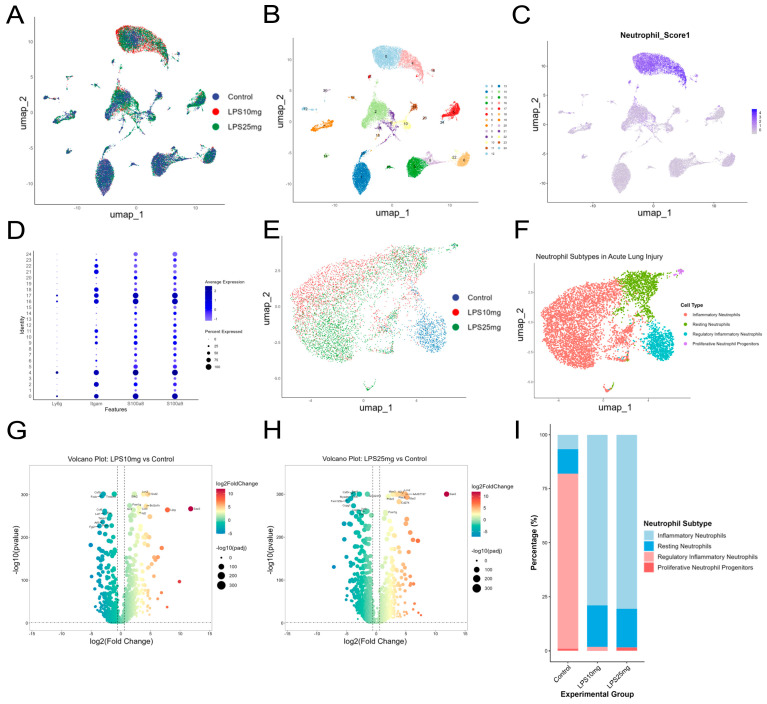
Single-cell transcriptomic analysis reveals neutrophil heterogeneity and inflammation-associated changes in ALI. (**A**) UMAP by treatment group. (**B**) UMAP by clusters. (**C**) Neutrophil module scoring. (**D**) Bubble plot showing the distribution of Ly6g, Itgam, S100a8, and S100a9 expression across clusters. (**E**) UMAP plot of neutrophil subtypes across treatment groups. (**F**) UMAP plot of neutrophil subtype annotation. (**G**) Volcano plot of LPS 10 mg/kg vs. Control. (**H**) Volcano plot of LPS 25 mg/kg vs. Control, with dotted lines indicating the thresholds of *p* < 0.05 and |log_2_FC| > 0.585. (**I**) Stacked bar plot showing the distribution of neutrophil subtypes across treatment groups.

Figure 3A,B shows UMAP visualizations of single-cell transcriptomic profiles from mouse lung tissues in the control, LPS 10 mg/kg, and LPS 25 mg/kg groups. A total of 25 transcriptionally distinct clusters were identified through unsupervised clustering, providing a foundation for downstream immune cell type annotation and differential expression analysis. To identify neutrophils within the lung single-cell transcriptomic dataset, we calculated a neutrophil signature score based on the expression of canonical markers including Ly6g, Itgam, S100a8, and S100a9 [27]. As shown in Figure 3C, clusters 0 and 4 displayed the highest neutrophil scores, indicating strong neutrophil identity. To further validate this classification and eliminate potential confounding effects from doublets or ambiguous populations, we examined the overall gene expression patterns and cellular composition across clusters (Figure 3D), confirming clusters 0 and 4 as bona fide neutrophils (i.e., genuine neutrophil populations validated by canonical marker expression and gene expression profiles). Figure 3E,F shows neutrophils from clusters 0 and 4 reclustered and colored by treatment condition (Control, LPS 10 mg/kg, and LPS 25 mg/kg), illustrating a dose-dependent shift in neutrophil states with increasing LPS exposure. Subtype annotation identified four functionally distinct neutrophil populations: inflammatory neutrophils, resting neutrophils, regulatory inflammatory neutrophils, and proliferative neutrophil progenitors. These subtypes reveal the functional heterogeneity of neutrophils in acute lung injury, reflecting their roles in inflammation, immune regulation, and tissue repair across disease progression. Stacked bar plot showing the percentage of each neutrophil subtype (inflammatory, resting, regulatory inflammatory, and proliferative progenitors) in the Control, LPS 10 mg/kg, and LPS 25 mg/kg groups. In the control group, regulatory inflammatory neutrophils accounted for the majority of the population. Upon LPS stimulation, there was a marked expansion of inflammatory neutrophils and a concurrent reduction in regulatory subsets, suggesting a shift toward a proinflammatory state in acute lung injury [28] (Figure 3I).

Volcano plots showing differentially expressed genes in neutrophils following low- and high-dose LPS stimulation compared to the control group. LPS treatment induced robust transcriptional changes in neutrophils, with a dose-dependent increase in the number and magnitude of significantly upregulated genes. Notably, several genes consistently upregulated across both doses, including Saa3, Lcn2, Csf3, and Sod2, are well-known markers of inflammation and oxidative stress [29,30,31]. These findings highlight the conserved inflammatory gene signatures and stress responses activated in neutrophils during ALI progression (Figure 3G,H).

**Figure 4:**
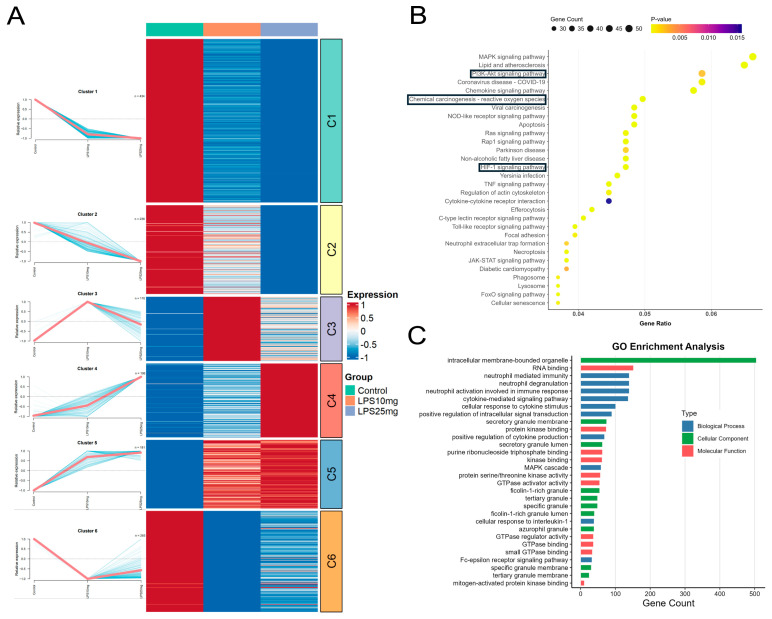
Single-cell transcriptomic analysis reveals neutrophil heterogeneity and inflammation-associated changes in ALI. (**A**) Mfuzz clustering of differentially expressed genes, with key pathways highlighted by black lines. (**B**) KEGG pathway enrichment of selected clusters. (**C**) GO term enrichment of selected clusters.

Differentially expressed genes in neutrophils across the Control, LPS 10 mg/kg, and LPS 25 mg/kg groups were subjected to soft clustering using the Mfuzz algorithm. Six distinct expression patterns were identified. Clusters C1, C2, C3, and C5, which exhibited LPS-responsive and dose-dependent dynamic trends, were selected for subsequent GO and KEGG pathway enrichment analyses (Figure 4A). Bubble plot showing KEGG pathway enrichment results for Clusters C1, C2, C3, and C5. Significantly enriched pathways include the PI3K-AKT signaling pathway, HIF-1 signaling pathway, and glutathione metabolism, all of which are closely associated with oxidative stress and inflammatory regulation in acute lung injury (Figure 4B). Bar plot showing GO enrichment results for genes in Clusters C1, C2, C3, and C5. The enriched terms include biological processes such as neutrophil-mediated immunity, degranulation, and cytokine signaling, as well as components related to granules and intracellular membranes, and molecular functions such as RNA binding and kinase activity. These results reflect the involvement of neutrophils in immune activation and stress signaling in response to LPS-induced ALI (Figure 4C).

### 2.4. Single-Cell Transcriptomic Analysis Reveals Macrophage Heterogeneity and Inflammation-Associated Changes in ALI

**Figure 5:**
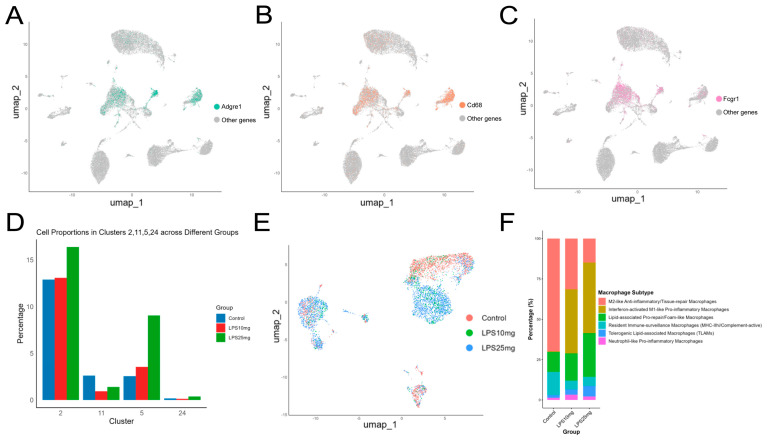
Identification and functional classification of macrophage populations in LPS-induced acute lung injury. (**A**) Bar plot based on cluster composition across groups. (**B**–**D**) UMAP feature plots displaying the expression of classical macrophage markers Adgre1, Cd68, and Fcgr1, predominantly enriched in Clusters 2, 5, 11, and 24. (**E**) UMAP visualization based on treatment group distribution. (**F**) Stacked bar plot based on annotated macrophage subtypes.

Based on Figure 5A–C, the classical macrophage marker genes Adgre1, Cd68, and Fcgr1 were mapped onto the UMAP, showing high expression predominantly in Clusters 2, 5, 11, and 24. As shown in the cluster proportion analysis (Figure 5D), Clusters 2, 5, and 11 exhibited a consistent trend of increased cell proportions following LPS treatment. Therefore, Clusters 2, 5, and 11 were ultimately identified as macrophage populations and selected for subsequent functional analyses. Figure 5E shows the UMAP visualization of macrophage subtypes across different treatment groups, including Control, LPS 10 mg/kg, and LPS 25 mg/kg. Figure 5F presents the annotation results identifying six macrophage subpopulations with distinct functional characteristics, including M2-like anti-inflammatory/tissue-repair macrophages, interferon-activated M1-like pro-inflammatory macrophages, lipid-associated pro-repair/foam-like macrophages, and neutrophil-like pro-inflammatory macrophages. Notably, LPS stimulation led to a marked reduction in M2-like macrophages, accompanied by a dose-dependent increase in M1-like and foam-like macrophage subsets, suggesting pronounced macrophage polarization during the progression of ALI [32].

**Figure 6:**
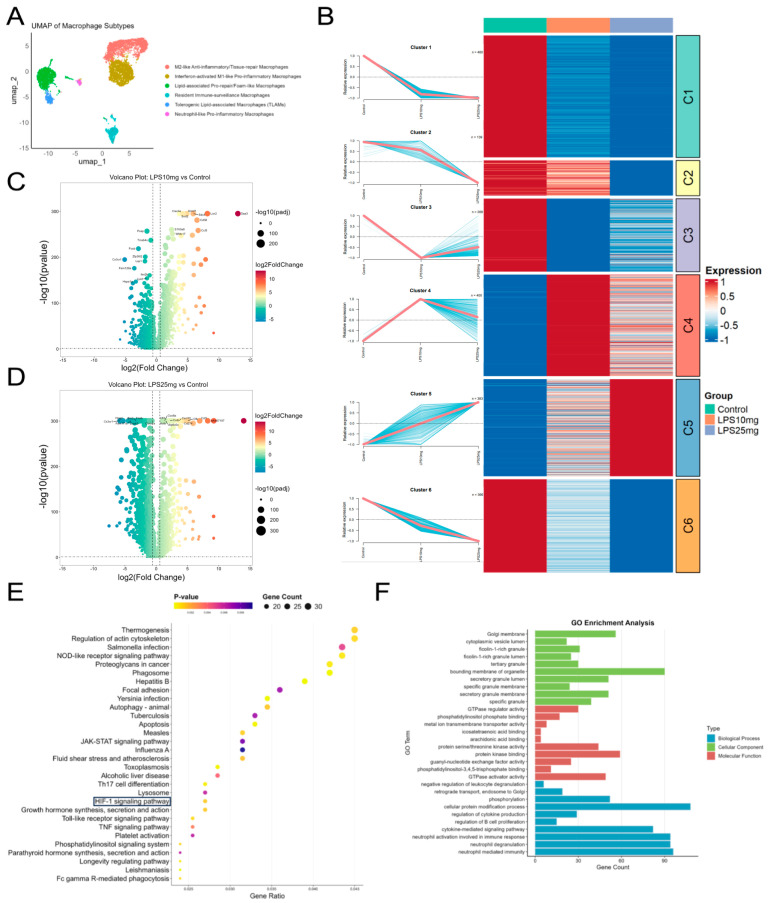
Functional clustering and enrichment analysis of macrophage transcriptional responses to LPS. (**A**) UMAP based on macrophage subtype classification. (**B**) Mfuzz clustering heatmap based on differentially expressed genes. (**C**,**D**) Volcano plots based on LPS 10 mg/kg and 25 mg/kg vs. Control, with dotted lines indicating the thresholds of *p* < 0.05 and |log_2_FC| > 0.585. (**E**) KEGG enrichment based on Mfuzz-selected gene clusters, with key pathways highlighted by black lines. (**F**) GO enrichment based on Mfuzz-selected gene clusters.

Based on the differentially expressed genes in macrophages, we performed Mfuzz clustering analysis and identified six distinct expression patterns. Among them, clusters 1, 2, 5, and 6 exhibited dynamic expression trends closely associated with LPS-induced lung injury and its dose-dependent progression. These clusters were selected for subsequent KEGG and GO enrichment analyses to explore the biological pathways and functions underlying macrophage responses in acute lung injury (Figure 6A,B). To further dissect the macrophage-specific transcriptional changes in ALI, we performed differential gene expression analysis between control and LPS-treated groups within the macrophage population. As shown in Figure 6C,D, volcano plots highlight numerous differentially expressed genes in response to 10 mg/kg and 25 mg/kg LPS. Notably, genes such as Cd38, Clec4e, and S100a8, which are associated with inflammation and oxidative stress, were significantly upregulated in both LPS-treated groups. These genes were also identified in the proteomic datasets, reinforcing their potential role in mediating macrophage-driven inflammatory responses during ALI progression [33,34,35]. Based on the differentially expressed genes from the selected macrophage clusters (C1, C2, C5, and C6), KEGG and GO enrichment analyses were performed to explore their potential biological functions. As shown in Figure 6E, KEGG pathway analysis revealed enrichment in pathways related to inflammation, oxidative stress, and immune response, including the HIF-1 signaling pathway, TNF signaling pathway, Toll-like receptor signaling, lysosome, and Fc gamma R-mediated phagocytosis. These pathways are closely associated with macrophage activation, pro-inflammatory signaling, and pathogen clearance. The GO enrichment results (Figure 6F) further indicated significant involvement in processes such as cytokine-mediated signaling, immune effector regulation, leukocyte activation, and intracellular vesicle transport, suggesting that these macrophage subsets play key roles in the inflammatory regulation during acute lung injury. Integrating the findings from single-cell transcriptomic analyses of neutrophils and macrophages, together with proteomic data from whole blood and lung tissue, we observed that the HIF-1 signaling pathway—particularly the HIF-1α/VEGF axis—spans across systemic, tissue, and cellular levels. Notably, the PI3K/AKT pathway also appeared recurrently in these datasets. Based on these converging multi-omics results, we focused subsequent investigations on the HIF-1 signaling pathway, with particular attention to its interplay with oxidative stress and the PI3K/AKT cascade, and designed in vitro and in vivo experiments to validate the mechanistic role of Harpagide in modulating these pathways.

### 2.5. Histological and Oxidative Stress Evaluation of Lung Tissues in LPS-Induced Acute Lung Injury Model

**Figure 7:**
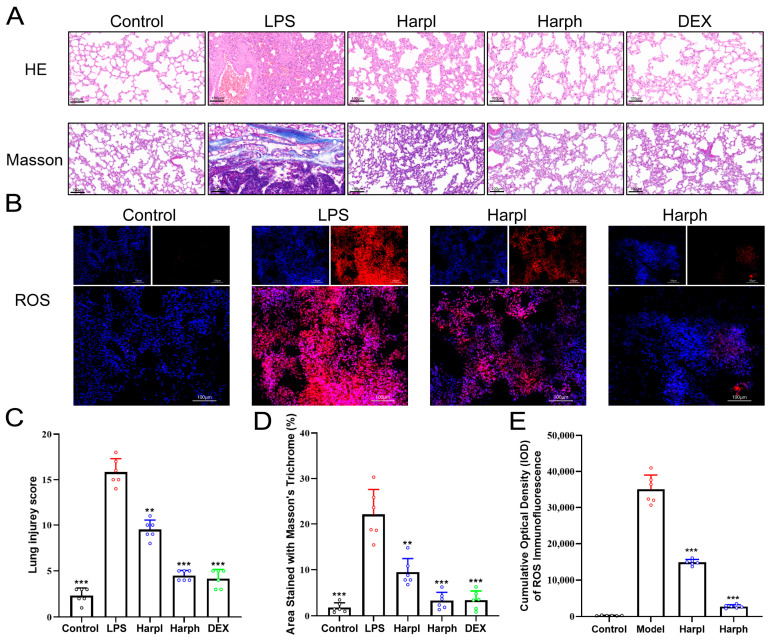
Histological and oxidative stress evaluation of lung tissues in LPS-induced acute lung injury model. The bar chart shows group colors: black = Control, red = LPS, blue = Harpagide (Harp), and green = Dexamethasone (DEX). (**A**) Representative HE and Masson’s trichrome staining of lung tissues from Control, LPS, HarpL, HarpH, and DEX groups. (**B**) Immunofluorescence staining of ROS in lung tissues, showing DAPI (blue) and ROS signals (red). (**C**) Lung injury scores based on HE staining (*n* = 6). (**D**) Fibrotic area quantification from Masson staining (*n* = 6). (**E**) Cumulative optical density (IOD) analysis of ROS fluorescence intensity (*n* = 6). Data are presented as mean ± SEM. ** *p* < 0.01, *** *p* < 0.001 vs. LPS group.

To evaluate the protective effects of Harpagide on ALI, histological and immunofluorescence analyses were performed. HE and Masson’s trichrome staining (Figure 7A) revealed that the LPS group exhibited marked alveolar wall thickening, inflammatory infiltration, and collagen deposition compared to the Control group. Treatment with HarpL or HarpH of Harpagide significantly alleviated these pathological changes, showing comparable efficacy to the positive control DEX. Quantitative analyses confirmed that Harpagide significantly reduced the lung injury score and fibrotic area (Figure 7C,D). In addition, ROS levels were evaluated by immunofluorescence staining (Figure 7B), which demonstrated massive oxidative stress in the LPS group. Both HarpL and HarpH treatment markedly reduced ROS accumulation in lung tissues, with HarpH achieving a more pronounced effect. Quantification of cumulative optical density (IOD) of ROS signals confirmed these observations (Figure 7E), suggesting that Harpagide exerts protective effects against ALI by suppressing oxidative stress and inflammation.

### 2.6. Harpagide Attenuates Inflammation and Oxidative Stress Through Modulation of Cytokine Levels and Redox-Related Signaling Pathways in LPS-Induced Acute Lung Injury

Based on previous multi-omics integrative analyses at the cellular, single-cell, and tissue proteomic levels, we found that the HIF-1 signaling pathway and oxidative stress responses were persistently activated within the immune microenvironment of macrophages and neutrophils. Consistent and significant enrichment of this pathway was also observed in both whole blood and lung tissue proteomics, suggesting that HIF-1α and oxidative stress signaling constitute a shared regulatory mechanism spanning cellular, tissue, and systemic levels. Considering the close relationship between HIF-1α and oxidative stress, we further investigated its role in the regulation of key oxidative stress–related factors, including Nrf2, HO-1, and VEGF.

**Figure 8:**
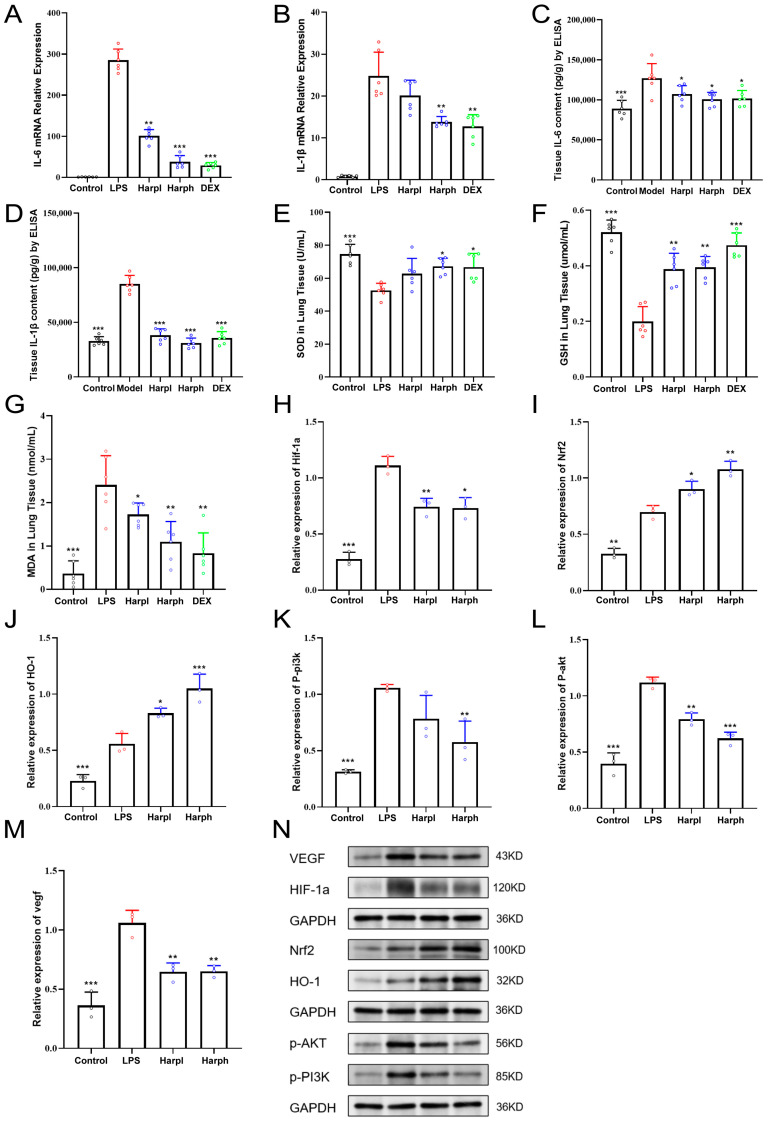
Harpagide attenuates inflammation and oxidative stress through modulation of cytokine levels and redox-related signaling pathways in LPS-induced acute lung injury. The bar chart shows group colors: black = Control, red = LPS, blue = Harpagide (Harp), and green = Dexamethasone (DEX). (**A**,**B**) mRNA expression of IL-6 and IL-1β in lung tissues measured by qPCR (*n* = 6). (**C**,**D**) IL-6 and IL-1β protein levels in lung tissues measured by ELISA (*n* = 6). (**E**–**G**) Quantification of oxidative stress markers: SOD and GSH levels, and lipid peroxidation product MDA. (**H**–**M**) Relative expression levels of key molecules in oxidative stress and cell survival signaling pathways, including Hif-1α, Nrf2, HO-1, p-AKT, p-PI3K, and VEGF (*n* = 3). (**N**) Quantification of protein expression levels of Hif-1α, Nrf2, HO-1, p-AKT, p-PI3K, and VEGF by densitometric analysis of Western blot bands. Data are presented as mean ± SEM. * *p* < 0.05, ** *p* < 0.01, *** *p* < 0.001 vs. LPS group.

To further investigate the anti-inflammatory effects of Harpagide, we measured the expression levels of key pro-inflammatory cytokines in lung tissues. As shown in Figure 8A,B, LPS significantly increased the mRNA expression of IL-6 and IL-1β compared to the Control group. Treatment with Harpagide at both low (HarpL) and high (HarpH) doses markedly reduced these elevations in a dose-dependent manner, with effects comparable to the positive control DEX. Consistently, ELISA results demonstrated that tissue protein levels of IL-6 and IL-1β were significantly elevated in the LPS group, while Harpagide treatment significantly suppressed cytokine production (Figure 8C,D). These findings indicate that Harpagide alleviates LPS-induced pulmonary inflammation by downregulating the expression and secretion of pro-inflammatory cytokines. To assess the antioxidant capacity of Harpagide in LPS-induced lung injury, levels of oxidative stress markers including SOD, GSH, and MDA were measured in lung tissues. As shown in Figure 8E–G, LPS exposure significantly decreased SOD and GSH levels while markedly increasing MDA content, indicating severe oxidative damage. Treatment with Harpagide significantly restored antioxidant levels (SOD and GSH) and reduced lipid peroxidation (MDA) in a dose-dependent manner. Notably, the HarpH group exhibited greater efficacy in restoring redox balance, comparable to that of DEX. These findings suggest that Harp mitigates oxidative stress during acute lung injury by enhancing endogenous antioxidant defenses and inhibiting oxidative damage.

To further explore the molecular mechanisms by which Harpagide exerts its antioxidant effects, we examined the expression of key regulators involved in oxidative stress and cell survival signaling pathways in lung tissues. As shown in Figure 8H–N, LPS stimulation significantly upregulated the expression of Hif-1α, p-AKT, p-PI3K, and VEGF. Harpagide treatment, particularly at the high dose HarpH, effectively reversed these changes. Meanwhile, the antioxidant-related factors Nrf2 and HO-1 were upregulated as a self-protective response. Notably, Harpagide further enhanced the expression of Nrf2 and HO-1, suggesting the activation of the Nrf2/HO-1 antioxidant pathway. In addition, Harpagide suppressed the excessive activation of the PI3K/AKT/Hif-1α signaling cascade, as evidenced by decreased expression levels of p-AKT, p-PI3K, and downstream VEGF.

### 2.7. Harpagide Alleviates Oxidative Stress by Downregulating HIF-1α and Activating the Nrf2/HO-1 Pathway in LPS-Induced A549 Cells

**Figure 9:**
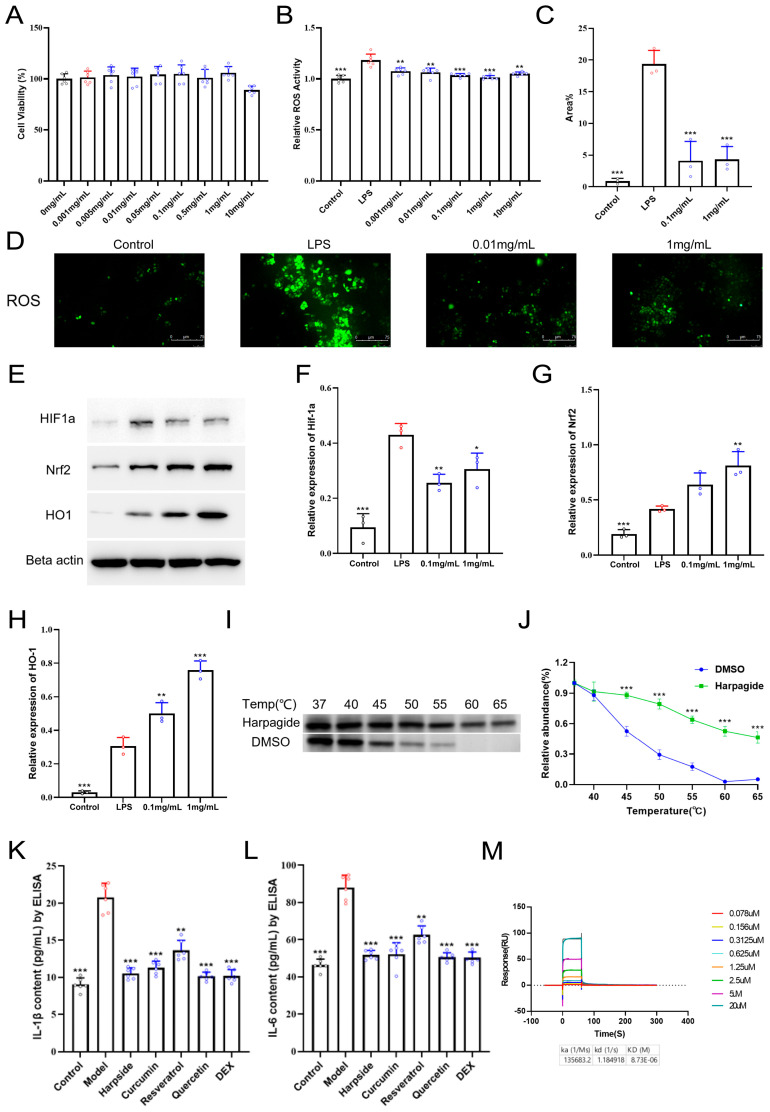
Harpagide alleviates oxidative stress by downregulating HIF-1α and activating the Nrf2/HO-1 pathway in LPS-induced A549 cells. (**A**) Cell viability assessed by the CCK-8 assay after 24 h Harpagide treatment at various concentrations (0–10 mg/mL). (**B**) Intracellular ROS levels measured using the DCFH-DA probe. (**C**) Quantification of ROS-positive area (%) based on immunofluorescence images. (**D**) Representative fluorescence images showing intracellular ROS distribution (green) in control, LPS-treated, and Harpagide-treated cells (100×). (**E**) Western blot analysis of HIF-1α, Nrf2, HO-1, and β-actin protein expression. (**F**–**H**) Densitometric quantification of HIF-1α (**F**), Nrf2 (**G**), and HO-1 (**H**) protein levels (*n* = 3). (**I**) CESTA analysis of HIF-1α stability with or without Harpagide treatment at 37–65 °C. (**J**) Quantification shows that Harpagide improves HIF-1α thermal stability compared to DMSO. (**K**,**L**) Comparison of anti-inflammatory effects of Harpagide with curcumin, resveratrol, quercetin, and DEX in LPS-stimulated RAW 264.7 macrophages; secretion levels of IL-1β (**K**) and IL-6 (**L**) were measured by ELISA (*n* = 6). (**M**) SPR analysis of Harpagide binding to HIF-1α, showing concentration-dependent sensorgrams and kinetic parameter determination. Data are presented as mean ± SEM. * *p* < 0.05, ** *p* < 0.01, *** *p* < 0.001 vs. LPS or DMSO group.

As shown in Figure 9A, Harpagide exhibited no cytotoxicity toward A549 lung epithelial cells at concentrations ranging from 0.001 to 1 mg/mL, as determined by the CCK-8 assay. Notably, a decline in cell viability was only observed at the highest concentration of 10 mg/mL, suggesting that Harpagide maintains a high safety margin and cellular compatibility within the effective concentration range. In Figure 9B, intracellular ROS levels were significantly elevated in the LPS group compared to the control, whereas Harpagide treatment markedly reduced ROS accumulation in a dose-dependent manner. Among all tested concentrations, 0.1 mg/mL and 1 mg/mL showed the most pronounced inhibitory effects on ROS production, which was further confirmed by quantitative fluorescence area analysis (Figure 9C). Based on these observations, 0.1 mg/mL and 1 mg/mL were selected as representative doses for subsequent ROS immunofluorescence experiments. As illustrated in Figure 9D, cells treated with Harpagide at these concentrations displayed a dramatic reduction in green ROS fluorescence compared to the LPS-stimulated LPS group, indicating effective alleviation of oxidative stress.

Furthermore, we compared the anti-inflammatory efficacy of Harpagide with three representative phytochemicals, curcumin, resveratrol, and quercetin, which have all been reported to confer protective effects in LPS of acute lung injury. Using the murine macrophage cell line RAW 264.7, cells in each experimental group were treated with 1 μmol of the respective compound, followed by LPS stimulation to induce an inflammatory response. The secretion levels of IL-1β and IL-6 were subsequently quantified by ELISA. As shown in Figure 9K,L, Harpagide markedly suppressed the production of these pro-inflammatory cytokines, with inhibitory effects comparable to those of curcumin, resveratrol, quercetin, and the positive control DEX (Figure 9K,L).

As shown in Figure 9E–H, Western blot analysis revealed that LPS stimulation markedly upregulated the expression of HIF-1α while slightly increasing the levels of Nrf2 and HO-1 in A549 cells. In contrast, Harpagide treatment significantly attenuated LPS-induced HIF-1α expression and robustly enhanced the protein levels of Nrf2 and HO-1 in a dose-dependent manner, with the 1 mg/mL group showing the most pronounced effect. These results suggest that Harpagide not only inhibits pro-inflammatory HIF-1α activation but also promotes the antioxidant response mediated by the Nrf2/HO-1 axis. To further evaluate the binding interaction between Harpagide and HIF-1α, a CESTA was performed. As shown in Figure 9I,J, increasing the temperature from 37 °C to 65 °C led to progressive degradation of HIF-1α protein in the DMSO-treated group, indicating thermal instability. However, in the presence of Harpagide, the thermal stability of HIF-1α was markedly enhanced, as evidenced by significantly higher residual protein levels across all temperature points. SPR analysis directly quantified the interaction between Harpagide and HIF-1α. As shown in the newly added figure, Harpagide bound to HIF-1α in a concentration-dependent manner, with a dissociation constant (K_D_) of 8.73 μM, where K_D_ represents the equilibrium constant describing the ratio of the dissociation to association rates. The association rate constant (k_a_) was 1.36 × 10^5^ M^−1^ s^−1^, reflecting the speed at which the complex forms, and the dissociation rate constant (k_d_) was 1.18 s^−1^, indicating the rate at which the complex dissociates. These parameters collectively indicate that this small molecule–protein interaction exhibits a moderate-to-strong binding affinity. These result supports the notion that Harpagide directly binds to and stabilizes HIF-1α, consistent with the MD simulation findings and indicative of a specific ligand–target interaction. Taken together, these data confirm that Harpagide exerts a dual regulatory effect by downregulating LPS-induced HIF-1α expression while enhancing Nrf2-mediated antioxidant defense, and that it likely achieves this through direct binding to HIF-1α, increasing its structural stability under thermal stress (Figure 9M).

### 2.8. Harpagide Stably Binds to the Region of HIF-1α and Enhances Complex Stability During MD Simulation

To further validate the binding interaction between Harpagide and HIF-1α, we conducted molecular docking and MD simulations. Molecular docking is a computational approach that predicts ligand–receptor interactions by evaluating energy compatibility, spatial fitting, and chemical complementarity. In this study, Harpagide was initially placed at a random position at a certain distance from the surface of HIF-1α, without any predefined binding site. During the simulation, spontaneous conformational evolution and molecular interactions were observed in the absence of any external bias to determine whether Harpagide could stably bind to HIF-1α, thereby reflecting its potential as a targeted ligand.

**Figure 10:**
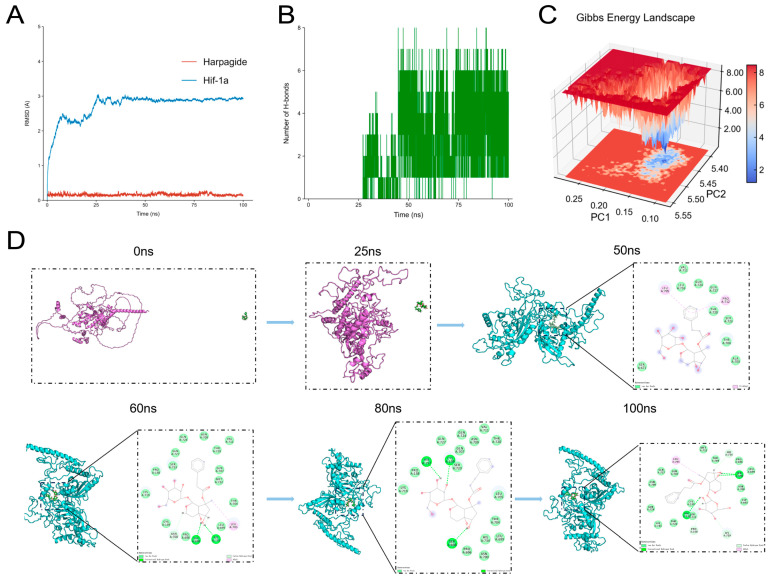
Harpagide stably binds to the region of HIF-1α and enhances complex stability during MD simulation. (**A**) RMSD analysis of the HIF-1α–Harpagide complex over 100 ns. (**B**) Hydrogen bond count between Harpagide and HIF-1α during the simulation. (**C**) Gibbs free energy landscape showing a stable low-energy binding conformation after 50 ns. (**D**) Representative hydrogen bond interactions between Harpagide and residue.

To explore the dynamic behavior of the HIF-1α–Harpagide complex, we performed a 100-nanosecond (ns) MD simulation, assessing complex stability through root mean square deviation (RMSD) and hydrogen bond fluctuations. RMSD, measured in angstroms (Å, 1 Å = 10^−10^ m), reflects structural deviation over time, with lower values indicating more stable conformations. Trajectory analysis revealed that after 50 ns, the relative position between Harpagide and HIF-1α remained stable, with no significant displacement observed, and the complex maintained a stable configuration throughout the remaining simulation (Figure 10A).

Hydrogen bonding also plays a key role in stabilizing ligand–receptor interactions. As shown in Figure 10B, the number of hydrogen bonds between Harpagide and HIF-1α remained relatively constant after 50 ns, averaging approximately three bonds, with a peak of up to eight bonds at certain time points—further supporting the persistence of the interaction.

The Gibbs free energy landscape provides insight into the stability of the receptor–ligand complex. Blue and purple regions on the landscape indicate low-energy, stable conformational states. If the protein–ligand interaction is weak, the energy landscape typically shows multiple rugged local minima; in contrast, strong and stable interactions yield a single, smooth energy basin. As shown in Figure 10C, the three-dimensional Gibbs free energy landscape of the HIF-1α–Harpagide complex after 50 ns reveals a single, sharply defined low-energy region, indicating a highly stable binding conformation.

To visualize the ligand’s behavior within the binding pocket, we extracted structural snapshots of the HIF-1α–Harpagide complex at 0, 25, 50, 60, 80, and 100 ns. Results showed that starting from 50 ns, Harpagide stably localized within the binding region of HIF-1α spanning residues 600–800. Structurally, the C-terminal region of HIF-1α comprises the inhibitory domain (ID, residues 576–785) and the C-terminal transactivation domain (C-TAD, residues 786–826). The ID can modulate the activity of C-TAD by influencing its conformational exposure and is considered a potential pharmacological target for regulating HIF-1α stability and function. In contrast, the C-TAD is a critical region for binding co-activators such as CBP/p300 and mediating transcriptional activation of HIF target genes. Notably, Asn803 within the C-TAD is a hydroxylation site for FIH (factor inhibiting HIF), and its modification prevents CBP/p300 binding, thereby modulating HIF-1α’s transcriptional activity. This may partially explain the mechanism by which Harpagide exerts a regulatory effect on HIF-1α function [36].

This stable binding pattern was further maintained at 60, 80, and 100 ns. Remarkably, at these time points, residue 698 consistently formed stable hydrogen bonds with Harpagide (Figure 10D), highlighting its potential structural relevance in mediating ligand interaction within the inhibitory domain of HIF-1α.

## 3. Discussion

ALI is a complex pathological condition triggered by various insults, among which LPS is a well-established inducer. LPS activates the innate immune system and elicits a robust systemic inflammatory response syndrome, leading to pulmonary tissue injury, impaired gas exchange, and massive infiltration of immune cells. Although traditionally viewed as a localized pulmonary process, ALI is increasingly recognized as a systemic immune disorder characterized by widespread immune cell activation and trafficking. Current research and clinical interventions have primarily focused on local tissue injury or employed lung proteomics to elucidate underlying mechanisms. While these approaches have provided critical insights, they fall short of capturing the full spectrum of immune dysregulation observed in ALI [37].

In clinical settings, standard therapeutic strategies often fail to effectively address ALI-associated complications, including secondary infections, immunosuppression, and multi-organ dysfunction, which contribute to poor patient outcomes. This highlights the limitations of locally centered investigations and underscores the need for systemic-level studies that integrate peripheral immune and fluid biomarkers to uncover dynamic disease mechanisms and potential therapeutic targets [38].

In this study, we performed both lung tissue and Whole blood proteomic analyses in an LPS-induced ALI LPS. While lung proteomics revealed localized changes, the Whole blood proteome reflected systemic immune dynamics, particularly involving neutrophil extracellular trap formation, chemotaxis pathways, and macrophage-associated phagocytic and inflammatory signaling. These findings indicate that neutrophils and macrophages play pivotal roles in mediating the systemic inflammatory response in ALI.

To further delineate the functional heterogeneity and phenotypic states of these immune cells within the lung microenvironment, we employed scRNA-seq to construct a high-resolution atlas of immune cell populations. Integrative analysis of proteomic and transcriptomic data revealed persistent activation of oxidative stress-related pathways, notably the HIF-1α, Nrf2/HO-1 and PI3K/AKT signaling cascades, across both bulk and single-cell levels. Oxidative stress not only directly promotes immune cell activation and proinflammatory cytokine release but also exacerbates lung injury through metabolic and signaling alterations.

Mechanistically, the HIF-1α, PI3K–AKT, and Nrf2/HO-1 signaling pathways exhibit complex bidirectional regulation and cross-activation. Hypoxia or activation of the PI3K–AKT cascade promotes the synthesis and stabilization of HIF-1α, which in turn activates Nrf2 via induction of VEGF/VEGFR2 signaling, thereby upregulating HO-1 expression [39]. HIF-1α, the master regulator of cellular responses to hypoxia, directly binds to the promoter regions of inflammatory cytokines and enhances their transcriptional activity. Previous studies have shown that HIF-1α not only amplifies IL-1β expression through inflammasome-related mechanisms, but also engages hypoxia response elements within the IL-6 promoter, thereby markedly increasing IL-6 transcription and secretion [40,41]. Nrf2 can also stabilize HIF-1α by binding to antioxidant response elements (AREs) within the HIF1A promoter or through downstream effectors such as NQO1, TRX1, and HO-1–derived carbon monoxide, forming a positive feedback loop [42,43]. However, the antioxidant effects mediated by Nrf2/HO-1 can lower intracellular ROS levels, restore PHD–VHL–dependent degradation of HIF-1α, and thus exert negative regulation [44]. Collectively, these interactions constitute a dynamic network governing inflammatory responses and oxidative stress, with the direction of regulation determined by cell type, degree of hypoxia, and redox status. Analysis of published clinical datasets further supports our experimental findings. In a recent study of ARDS patients, serum HIF-1α and VEGF levels were significantly higher in individuals with poor prognosis than in those with favorable outcomes, and logistic regression identified both markers as independent predictors of disease severity (HIF-1α OR = 3.885; VEGF OR = 4.204; *p* < 0.05). These clinical results are consistent with our murine and single-cell analyses, which revealed activation of the HIF-1α/VEGF axis and associated PI3K/AKT signaling in injured lung tissue [45].

To evaluate the regulatory role of Harpagide in the pathological cascade of acute lung injury, we systematically examined its protective effects in both in vivo and in vitro settings. Our results demonstrate that Harpagide markedly ameliorates LPS-induced lung tissue injury and fibrosis, restores redox homeostasis by enhancing antioxidant enzyme activity and reducing lipid peroxidation, and significantly suppresses the production of pro-inflammatory cytokines, thereby attenuating pulmonary inflammation. At the signaling level, Harpagide inhibits hyperactivation of the HIF-1α and PI3K/AKT pathways while upregulating the Nrf2/HO-1 antioxidant defense axis, suggesting a dual regulatory effect on oxidative stress and inflammation through multi-pathway coordination. These effects were further validated in vitro in lung epithelial cells and macrophages, indicating that Harpagide directly modulates immune cell activation and inflammatory mediator release. Target engagement studies using SPR and CETSA confirmed direct binding of Harpagide to HIF-1α, and molecular dynamics simulations revealed a stable binding conformation within the inhibitory/C-terminal transactivation domain of HIF-1α, implying that this interaction may influence the structural stability and transcriptional activity of HIF-1α.

In conclusion, our integrative single-cell transcriptomic and proteomic analysis highlights the central roles of neutrophils and macrophages in ALI pathogenesis and reveals the pathogenic relevance of HIF-1α signaling in oxidative stress regulation. Harpagide emerges as a promising therapeutic candidate capable of rebalancing immune-metabolic dysregulation through modulation of these key pathways.

## 4. Methods, Materials, and Animals

### 4.1. Chemicals and Reagents

Harpagide (HPLC ≥ 98%; Cat. No. A0335) was purchased from Chengdu Manster Biotechnology Co. (Chengdu, China). A549 lung epithelial cells (Cat. No. 164210), RAW 264.7 murine macrophage cells (Cat. No. CL-0190), and RPMI-1640 medium (Cat. No. PM150110B) were all obtained from Procell Life Science & Technology Co., Ltd. (Wuhan, China). Commercial assay kits for glutathione (GSH; Cat. No. RXWB0113-96), superoxide dismutase (SOD; Cat. No. RXWB0482-96), and malondialdehyde (MDA; Cat. No. RXWB0005-96) were obtained from Ruixin Biotechnology Co. (Quanzhou, China), while enzyme-linked immunosorbent assay (ELISA) kits for interleukin-6 (IL-6; Cat. No. SYP-M0031) and interleukin-1β (IL-1β; Cat. No. SYP-M0026) were acquired from Youpin Biotechnology Co. (Wuhan, China). Antibodies against vascular endothelial growth factor (VEGF, 66828-1-Ig), hypoxia-inducible factor 1-alpha (HIF-1α, 209601-1-Ig), phosphorylated protein kinase B (p-AKT, 66444-1-Ig), phosphorylated phosphoinositide 3-kinase (p-PI3K, 17366-1-AP), nuclear factor erythroid 2-related factor 2 (NRF2, 16396-1-AP), heme oxygenase 1 (HO-1, 10701-1-AP), and β-actin (66009-1-Ig), as well as an antibody for the immunofluorescent detection of ROS, were used in this study. The p-PI3K antibody was purchased from Cell Signaling Technology (Danvers, MA, USA), and all other antibodies were obtained from Proteintech (Wuhan, China). Recombinant human HIF-1α protein was purchased from Wuhan Huamei Biotech Co., Ltd. (Wuhan, China; catalog no. CSB-EP624113HU).

### 4.2. Animal Model of Acute Lung Injury

Male C57BL/6 mice (20–25 g, 8–10 weeks old) were obtained from the Peking University Health Science Center (Beijing, China). All animal procedures were conducted in accordance with the National Institutes of Health Guide for the Care and Use of Laboratory Animals and were approved by the Institutional Review Board (or Ethics Committee) of the China Academy of Chinese Medical Sciences (Approval No. Approval No. ERCCACMS21-2208-03, Approval Date: 9 August 2022). Mice were housed under standard specific pathogen-free (SPF) laboratory conditions (22 ± 1 °C, 55 ± 5% humidity, 12 h light/dark cycle) with ad libitum access to standard chow and water. Animals were randomly assigned to experimental groups using a random number generator, and all outcome assessments and data analyses were performed by investigators blinded to group allocation to minimize bias.

Lipopolysaccharide (LPS, from *Escherichia coli* O111:B4) was purchased from Sigma-Aldrich (St. Louis, MO, USA). A total of 60 mice were randomly divided into five groups (*n* = 12 per group): normal control, LPS model, dexamethasone (DEX, 2 mg/kg), low-dose Harpagide (HarpL, 40 mg/kg), and high-dose Harpagide (HarpH, 80 mg/kg). Except for the control group, all mice received an intraperitoneal injection of LPS (10 mg/kg) to induce an acute inflammatory model [46]. One hour later, the DEX, HarpL, and HarpH groups were administered the corresponding treatments intraperitoneally. Mice were euthanized 12 h after LPS injection, and tissue samples were collected for subsequent analyses.

### 4.3. Lung Tissue Proteomics Analysis

Protein concentrations were quantified at 280 nm using a NanoDrop spectrophotometer (Thermo Scientific, Waltham, MA, USA) with an extinction coefficient of 1.1 AU. Sample preparation was performed using the filter-aided sample preparation (FASP) method to eliminate detergents and facilitate enzymatic digestion. In brief, 200 μL of UA buffer (8 M urea in 0.1 M Tris-HCl, pH 8.5) was added to YM-30 Microcon centrifugal filter units (Millipore, Billerica, MA, USA). Lung tissue protein samples were loaded and centrifuged at 14,000× *g* for 15 min at 20 °C, and this step was repeated twice. Subsequently, 50 μL of 0.05 M iodoacetamide in 8 M urea was added to the filters and incubated in the dark for 20 min. The filters were washed twice with 100 μL UA buffer, followed by three washes with 100 μL of 50 mM ammonium bicarbonate (NH_4_HCO_3_).

For tryptic digestion, 100 μL of 50 mM NH_4_HCO_3_ containing sequencing-grade trypsin (Promega, San Luis Obispo, CA, USA) was added to each filter at a protein-to-enzyme ratio of 100:1. The samples were incubated overnight at 37 °C, and peptides were collected by centrifugation at 14,000× *g* for 15 min at 20 °C.

Peptides were analyzed using an Orbitrap Fusion Lumos Tribrid mass spectrometer (Thermo Fisher Scientific) coupled with an EASY-nLC 1000 nano-LC system (Thermo Fisher Scientific). Chromatographic separation was achieved on a 10 cm reversed-phase C18 column (75 μm inner diameter) packed with 3 μm XB-C18 resin (Welch Materials, West Haven, CT, USA), using a linear gradient of 3–100% buffer B (99.5% acetonitrile, 0.5% formic acid) in buffer A (99.5% water, 0.5% formic acid) over 75 min, at a flow rate of 350 nL/min. The entire LC-MS/MS run, including sample loading and column washing, lasted approximately 90 min.

Electrospray ionization was performed at 2.0 kV. Data-dependent acquisition was employed with a dynamic exclusion window of 18 s. MS1 scans were acquired at a resolution of 70,000 with an AGC target of 3e6 and a maximum injection time of 20 ms. MS2 scans were collected at a resolution of 17,500 with an AGC target of 1e6 and a maximum injection time of 60 ms. The scan range was set to 300–1400 *m*/*z*, and the top 20 most intense precursor ions were selected for fragmentation. Raw MS data were processed using a database search strategy, and protein identification and quantification were conducted using R Studio software (version 4.3.3).

Differentially expressed genes (DEGs) were identified using the limma package (v3.58.1). Genes were considered significantly differentially expressed if they met the criteria of an adjusted *p*-value < 0.05 (Benjamini–Hochberg correction) and an absolute fold change (|FC|) > 1.5, where FC > 1.5 indicates upregulation and FC < 0.67 indicates downregulation [47].

### 4.4. Whole Blood Proteomics

Whole blood samples were incubated at room temperature with a binding buffer (50 mM Tris, 10 mM EDTA) and magnetic polystyrene microbeads. Following incubation, the mixtures were transferred to centrifuge tubes and placed on a magnetic rack to allow bead capture. The beads were then resuspended in fresh binding buffer, vortexed briefly, and repositioned on the magnetic rack for separation. This process was followed by three sequential washes using wash buffer, with magnetic separation and removal of the supernatant after each step. Subsequently, the bead-bound material was incubated sequentially with Lysis Buffer. Samples were then heated at 95 °C for 10 min to facilitate protein denaturation, followed by enzymatic digestion with trypsin for 2 h at room temperature. Upon completion of digestion, peptides were washed successively with Column Wash Buffers. After each wash, samples were magnetically separated and supernatants discarded. The final eluate was collected and stored for downstream proteomic analysis. DEGs were identified using the limma package (v3.58.1). Genes were considered significantly differentially expressed if they met the criteria of an adjusted *p*-value < 0.05 (Benjamini–Hochberg correction) and an absolute fold change (|FC|) > 1.5, where FC > 1.5 indicates upregulation and FC < 0.67 indicates downregulation.

### 4.5. Single-Cell Transcriptomic Analysis

A bioinformatic analysis was conducted using single-cell transcriptomic data from lung tissues obtained from the GEO database (GSM8209056), which includes control, 10 mg/kg LPS-treated, and 25 mg/kg LPS-treated groups, to investigate the role and underlying mechanisms of immune cells in an ALI model. Differentially expressed genes (DEGs) were identified using a threshold of adjusted *p*-value < 0.05 and absolute fold change (|FC|) > 1.5, where FC > 1.5 indicates upregulation and FC < 0.67 indicates downregulation. Raw data were processed using the Seurat package (v5.0) in the R environment. During quality control, cells with >10% mitochondrial gene content or fewer than 200 detected genes were excluded as low-quality cells. Batch effects across samples were corrected using the harmony (v0.1.1) algorithm. Dimensionality reduction was performed via PCA, and the top 30 principal components were used for downstream clustering analysis. Cell clusters were identified using the “FindNeighbors” and “FindClusters” functions with a resolution of 0.1, and visualized in two dimensions using the “RunUMAP” function. Further subpopulation analysis was performed to characterize neutrophils and macrophages Neutrophils were identified based on the expression of Ly6g, Itgam, S100a8, and S100a9, while macrophages were marked by Adgre1, Fcgr1, and Cd68. Cell type annotation was guided by the CellMarker 2.0 database and previously published literature, and further refined through evaluation of canonical marker gene expression within each cluster. Based on this integrative approach, neutrophils were further classified into four functional states. Inflammatory neutrophils exhibited high expression of Cxcl2, Cxcl3, Ccl3, Ccl4, Cd274, Il1rn, Sod2, Nfkbia, and Traf1. Resting neutrophils showed strong expression of Ly6g and Itgam, but low levels of activation- or progenitor-associated genes. Regulatory inflammatory neutrophils were defined by upregulation of Cd274 (PD-L1), MHC class II genes (H2-Aa, H2-Ab1), Tnip1, Batf, and Irf7, suggesting immunomodulatory potential. Proliferative neutrophil progenitors were characterized by high expression of Ly6c2, Cd117 (c-Kit), and cell cycle–related genes including Mki67, Top2a, Ube2c, Birc5, and Stmn1. Macrophages were similarly stratified into six phenotypic subtypes based on transcriptional signatures. M2-like anti-inflammatory/tissue-repair macrophages expressed Cd163, Mrc1 (Cd206), Arg1, Il10, and Trem2. Interferon-activated M1-like pro-inflammatory macrophages showed upregulation of Irf7, Stat1, Nos2, Tnf, Cxcl10, Cd86, and Cd80. Lipid-associated pro-repair or foam-like macrophages were marked by high expression of Trem2, Cd9, Cd36, Lpl, Fabp5, and Apoe. Resident immune-surveillance macrophages displayed elevated levels of MHC class II genes (H2-Aa, H2-Ab1, Cd74) and complement components (C1qa, C1qb, C1qc). Tolerogenic lipid-associated macrophages (TLAMs) co-expressed Cd9, Cd36, Trem2, Il10, and Slc40a1. Neutrophil-like pro-inflammatory macrophages were characterized by Trem1, S100a8, S100a9, Fcgr2b, and in some cases Ly6g or Mmp9. Final subtype assignments for both neutrophils and macrophages were supported by differential gene expression analysis, UMAP localization, and cluster-specific co-expression patterns, ensuring accurate annotation of immune heterogeneity within the dataset [48].

### 4.6. Histopathological Examination

In this study, a standardized histopathological scoring system was employed to evaluate lung injury in mice subjected to different treatment conditions. Lung tissues were fixed in 4% paraformaldehyde, followed by ethanol dehydration, paraffin embedding, sectioning, and hematoxylin and eosin (H&E) staining. Histological changes were examined under a light microscope, and high-resolution images were acquired using a digital slide scanner (Pannoramic 250, 3DHISTECH Ltd., Budapest, Hungary). Tissue sections were assessed at various magnifications to quantify key pathological features, including intra-alveolar fibrin deposition (hyaline membrane formation), alveolar hemorrhage (presence of red blood cells within alveolar spaces), vascular congestion (capillary engorgement within alveolar septa), alveolar wall thickening (thickness exceeding one cell layer), and leukocyte infiltration (number of inflammatory cells per field, 215 × 165 μm). Each feature was graded on a scale from 0 to 4, where 0 indicates no detectable change and 1 to 4 represent increasing severity across one to four quadrants per field. Leukocyte infiltration was scored based on cell counts within the defined area, ranging from fewer than 10 cells (score 0) to 75 or more cells (score 4). In addition, comprehensive histological assessment, including features such as alveolar collapse, hemorrhage, fibrin deposition, and vascular congestion, was used to determine the extent of lung injury. This scoring approach enabled quantitative comparison of pathological changes across experimental groups in the ALI model.

### 4.7. Masson’s Trichrome Staining

Paraffin-embedded tissue sections were deparaffinized in xylene (twice, 20 min each), followed by sequential immersion in 100% ethanol (twice, 5 min each), 75% ethanol (5 min), and rinsed in tap water. Sections were then incubated in potassium dichromate solution overnight and washed thoroughly with tap water. For nuclear staining, equal volumes of Iron Hematoxylin Solution A and B were mixed to prepare the working solution. Sections were stained in this solution for 3 min, rinsed with tap water, differentiated in acid alcohol, washed, and then blued in a bluing solution. After rinsing, sections were stained with Ponceau-acid fuchsin solution for 5–10 min, followed by a brief rinse in tap water. Slides were then incubated in phosphomolybdic acid solution for 3–5 min and transferred directly into aniline blue solution for 3–6 min without intermediate washing. Differentiation was performed using 1% glacial acetic acid, followed by dehydration with two changes of absolute ethanol. Finally, slides were cleared in xylene and mounted with a neutral resin. Stained sections were examined under a light microscope and images were captured for analysis. Collagen fibers appeared blue, while muscle fibers, fibrin, and red blood cells were stained red.

### 4.8. Immunofluorescence

Paraffin-embedded tissue sections were deparaffinized by sequential immersion in xylene I (15 min), xylene II (15 min), absolute ethanol I (5 min), and absolute ethanol II. The sections were then air-dried in a fume hood, rinsed briefly in tap water, and washed with distilled water. For antigen retrieval, the slides were placed in a retrieval box filled with pH 9.0 EDTA buffer and heated in a microwave oven (medium power for 8 min, pause for 8 min, followed by medium-low power for 10 min), ensuring that the buffer did not evaporate and the sections did not dry out. After natural cooling, the slides were washed three times in PBS (pH 7.4) on a decolorization shaker, 5 min each. To block endogenous peroxidase activity, the sections were incubated with 3% hydrogen peroxide for 15 min at room temperature in the dark, followed by three PBS washes. After gently drying the slides, a hydrophobic barrier was drawn around the tissue using a histochemical pen, and the sections were incubated with 3% BSA in PBS (or other blocking solution) for 30 min at room temperature. The primary antibody, diluted in antibody diluent, was then applied to the sections, which were incubated overnight at 4 °C in a humidified, light-protected chamber containing a small amount of water to prevent evaporation. After washing three times with PBS, HRP-conjugated secondary antibody, specific to the species of the primary antibody, was applied and incubated for 50 min at room temperature in the dark. Sections were washed again in PBS three times. For signal development, slides were incubated with tyramide-conjugated fluorescent dye (TYR488) for 10 min, followed by three PBS washes. Steps 2–7 were then repeated using a different tyramide dye for multiplex labeling. Finally, nuclei were counterstained with DAPI for 10 min in the dark after PBS washes, and slides were mounted using an anti-fade fluorescence mounting medium following three final PBS washes.

### 4.9. Quantification of Inflammatory Cytokines in Lung Tissue

Total RNA was extracted from lung tissue ground in liquid nitrogen using the FastPure Cell/Tissue Total RNA Isolation Kit V2 (Vazyme Biotech Co., Ltd., Nanjing, China) and quantified using a NanoDrop 2000 spectrophotometer (Thermo Fisher Scientific, USA). cDNA was synthesized using the M5 Super Plus qPCR RT Kit with gDNA Remover (Mei5 Bioservices Co., Ltd., Beijing, China) according to the manufacturer’s protocol. Quantitative real-time PCR (RT-qPCR) was performed using a 10 µL reaction system containing 5.0 µL of 2× Taq Pro Universal SYBR qPCR Master Mix (Vazyme Biotech Co., Ltd.), 0.2 µL each of the forward and reverse primers, 1 µL of cDNA, and 3.6 µL of nuclease-free water. The thermal cycling conditions were as follows: 95 °C for 30 s, followed by 40 cycles of 95 °C for 10 s and 60 °C for 30 s; a melting curve analysis was conducted at 95 °C for 15 s, 60 °C for 60 s, and 95 °C for 15 s. GAPDH was used as the internal reference gene, and the relative expression levels of IL-1β and IL-6 were calculated using the 2^−ΔΔCt^ method. Primer sequences are listed in Table 1.

### 4.10. Analysis of Antioxidant and Inflammatory Markers

Serum levels of IL-6 and IL-1β were measured using commercial ELISA kits according to the manufacturer’s instructions. Lung tissues were homogenized at 4 °C and centrifuged at 12,000 rpm for 10 min, and the resulting supernatants were collected for protein quantification. Levels of SOD, GSH, and MDA were determined using corresponding assay kits, following the provided protocols. For the in vitro comparison of anti-inflammatory efficacy, RAW 264.7 murine macrophages were pretreated with 1 μmol Harpagide, curcumin, resveratrol, or quercetin 1 h after LPS stimulation (1 μg/mL) and incubated for an additional 12 h. Culture supernatants were then collected, and IL-6 and IL-1β levels were quantified by ELISA using a microplate reader (Thermo Fisher Scientific, Waltham, MA, USA).

### 4.11. Western Blot Analysis (WB)

For tissue protein extraction, 25 mg of lung tissue was homogenized in 500 µL of ice-cold RIPA lysis buffer containing phosphatase inhibitors using a low-temperature tissue grinder for 2 min. The homogenate was incubated on ice with gentle shaking for 30 min and centrifuged at 12,000× *g* for 10 min at 4 °C. The supernatant was collected for further analysis.

For cell protein extraction, adherent cells were gently washed twice with ice-cold PBS to remove culture medium and serum proteins, and residual PBS was aspirated completely. Cells were then lysed directly on the plate using ice-cold RIPA buffer supplemented with protease and phosphatase inhibitors (typically 100–150 µL per well of a 6-well plate). The plates were incubated on ice for 30 min with occasional gentle shaking. Cell lysates were collected by scraping, transferred into tubes, and centrifuged at 12,000× *g* for 10 min at 4 °C to pellet debris. The supernatants were harvested for subsequent analysis.

Protein concentration was determined using a BCA protein assay kit according to the manufacturer’s instructions. Protein samples were mixed with 5× loading buffer at a 1:5 ratio and denatured at 95 °C for 8 min (65 °C for membrane proteins), then cooled on ice and stored at −80 °C. SDS-PAGE gels (6–15%) were prepared based on the molecular weight of the target proteins, and 24 µg of protein was loaded per lane. Electrophoresis was performed at a constant voltage of 80 V through the stacking gel, followed by 120 V through the resolving gel until the dye front reached the bottom. Proteins were transferred to PVDF membranes using the wet transfer method at 300 mA constant current, with transfer time determined by protein size (approximately 1 min per 1 kDa).

Membranes were blocked with 5% non-fat milk for 2 h at room temperature (5% BSA was used for phosphorylated proteins), followed by overnight incubation at 4 °C with primary antibodies including VEGF, HIF-1α, p-AKT, p-PI3K, NRF2, HO-1, GAPDH, and β-actin, diluted to their respective working concentrations. The next day, membranes were washed three times with TBST and incubated with HRP-conjugated secondary antibodies (1:10,000) for 1 h at room temperature, followed by another three TBST washes. Signals were visualized using enhanced chemiluminescence (ECL) and captured using an imaging system. For membrane reprobing, bound antibodies were removed using a stripping buffer, followed by rinsing and blocking for 2 h. Membranes were then re-incubated with the internal control antibody, and signals were detected using ECL.

### 4.12. Assessment of Cell Viability Using CCK-8 Assay

A549 lung epithelial cells were seeded in 96-well plates at a density of 1 × 10^4^ cells per well and allowed to adhere overnight. Cells were then stimulated with LPS (1 μg/mL) for 1 h, followed by treatment with the test compound at various concentrations (0, 0.001, 0.005, 0.01, 0.05, 0.1, 0.5, 1, and 10 mg/mL) for an additional 12 h. After drug incubation, cells were gently washed once with PBS to remove residual compound. Subsequently, 10 μL of CCK-8 reagent was added to each well containing 100 μL of fresh medium, and the plates were incubated at 37 °C for 1 h. Absorbance was measured at 450 nm using a microplate reader, and cell viability was calculated as a percentage relative to the untreated control group.

### 4.13. Detection of Intracellular ROS by Fluorescence Microplate Reader and Fluorescence Microscopy

A549 lung epithelial cells were seeded into black 96-well plates with transparent bottoms at a density of 1 × 10^5^ cells/mL and allowed to attach overnight. According to the experimental design, cells were stimulated with LPS (1 μg/mL) and/or treated with the test compound at various concentrations (0.001, 0.01, 0.1, 1, and 10 mg/mL) for 12 h. Intracellular ROS levels were measured using 2′,7′-dichlorodihydrofluorescein diacetate (DCFH-DA), diluted 1:1000 in RPMI-1640 medium. A total of 100 μL of the diluted probe was added to each well, and cells were incubated at 37 °C in the dark for 20 min. After incubation, cells were washed three times with DMEM/F12 medium to remove excess dye. Fluorescence intensity was recorded using a multifunctional microplate reader (excitation 488 nm, emission 525 nm). In parallel, cells seeded in 6-well plates underwent the same staining procedure, and ROS fluorescence distribution was visualized and imaged using a fluorescence microscope.

### 4.14. Cellular Thermal Shift Assay (CESTA)

To evaluate the binding of Harpagide to HIF-1α and its effect on protein thermal stability, a CESTA was performed. Cells in the logarithmic growth phase were seeded into culture dishes and incubated at 37 °C with 5% CO_2_ until reaching 70–80% confluence. The test group was treated with Harpagide (1 mg/mL) and incubated at 37 °C for 24 h, while the control group received an equal volume of DMSO with a final concentration of 0.1%. After treatment, cells were harvested and resuspended in PBS buffer containing 1 mmol/L PMSF. The cell suspension was aliquoted into PCR tubes corresponding to each temperature point and incubated at 37 °C, 40 °C, 45 °C, 50 °C, 55 °C, 60 °C, and 65 °C for 3 min to induce thermal denaturation. Immediately after heating, the tubes were transferred onto ice for 3 min. Cells were then resuspended in NP-40 lysis buffer and subjected to three freeze–thaw cycles using liquid nitrogen. Lysates were centrifuged at 20,000× *g* for 20 min at 4 °C. The supernatants were collected and mixed with an equal volume of 2× SDS loading buffer, denatured at 95 °C for 10 min, and subsequently analyzed by WB.

### 4.15. Surface Plasmon Resonance (SPR) Binding Assay

HIF-1α protein was immobilized on a CM5 sensor chip via amine coupling, and serial dilutions of Harpagide were prepared for kinetic analysis. Measurements were performed on a Biacore T200 instrument (Cytiva, Marlborough, MA, USA) using HBS-EP+ buffer containing 5% DMSO as the running buffer. Binding kinetics were recorded across a range of analyte concentrations, and data were analyzed using Biacore Evaluation Software (version 3.0). Sensorgrams were fitted to a 1:1 binding model to determine the association rate constant (k_a_) and dissociation rate constant (k_d_), and the equilibrium dissociation constant (K_D_) was calculated as the ratio of k_d_ to k_a_.

### 4.16. Molecular Dynamics Simulation

Molecular dynamics (MD) simulation is an important computational technique used to evaluate the binding affinity between small molecules and target proteins and to assess the stability of ligand–receptor complexes by monitoring their conformational changes over time. In this study, the interaction between HIF-1α and the natural product Harpagide was investigated using Gromacs 2020.03 with the CHARMM36-jul.ff force field. A 100-nanosecond (ns) MD simulation was conducted under near-physiological conditions. The structure of HIF-1α was predicted using the AlphaFold2 platform (https://www.alphafold.ebi.ac.uk/, accessed on 23 April 2025), and the three-dimensional structure of Harpagide was obtained from the PubChem database (https://pubchem.ncbi.nlm.nih.gov/, accessed on 23 April 2025). To closely mimic the natural recognition process under physiological conditions, Harpagide was initially placed randomly at a distance from the surface of HIF-1α without any predefined binding site. During the simulation, the spontaneous conformational evolution and molecular interactions were observed without external forces to determine whether Harpagide could stably bind to HIF-1α, thereby reflecting its potential targeting capability.

Harpagide was parameterized using AmberTools22 with the Generalized Amber Force Field. After hydrogenation, its electrostatic potential was calculated using Gaussian 16W via the Restrained Electrostatic Potential (RESP) method, and the resulting data were integrated into the system topology file. The system was solvated using the TIP3P water model, with the complex embedded in a water box ensuring a minimum distance of 1.2 nm (12 Å) between the outermost atoms of the protein and the box edges. To simulate physiological ionic strength, Na^+^ and Cl^−^ ions were added to achieve a final concentration of 0.154 M. Energy minimization was first performed using the steepest descent algorithm to eliminate steric clashes and optimize the initial conformation. This was followed by two phases of equilibration: first under an NVT ensemble (constant number of particles, volume, and temperature), gradually heating the system to 300 K while restraining solute positions; and then under an NPT ensemble (constant number of particles, pressure, and temperature), stabilizing the system at 300 K and 1 atm to ensure appropriate density and structural relaxation.

Subsequently, a 100 ns production MD simulation was carried out under near-physiological conditions. Trajectories were recorded at regular intervals for subsequent analysis of the binding dynamics of Harpagide, conformational changes in the complex, flexibility of the receptor structure, and key intermolecular interactions, providing mechanistic insights into the potential targeting of HIF-1α by Harpagide.

### 4.17. Statistical Analysis

The data were analyzed and visualized using GraphPad Prism version 9.4.0 and R software version 4.3.3. All results are expressed as mean ± standard deviation (M ± SD). One-way ANOVA was used for comparisons among multiple groups, while *t*-tests were performed for comparisons between two groups. Non-parametric tests were applied when appropriate.

## 5. Conclusions

This study provides a comprehensive multi-omics framework integrating single-cell transcriptomics and proteomics to elucidate the multilayered regulatory landscape of immune infiltration and microenvironmental disruption in acute lung injury. By identifying convergent activation of oxidative stress and inflammatory pathways, particularly HIF-1α, PI3K/AKT, and Nrf2/HO-1 signaling, we reveal shared molecular mechanisms across immune cell populations and tissue compartments. Harpagide, a natural compound, emerges as a potent modulator capable of one-drug, multilayer co-regulation, mitigating pulmonary damage by targeting these interconnected pathways. These findings offer mechanistic insight into ALI pathogenesis and position Harpagide as a promising candidate for multi-target therapeutic strategies in inflammatory lung diseases.

## Figures and Tables

**Table 1:** Primer sequence.

Primer Name	Sequence (5′ → 3′)
GAPDH F	CGACTTCAACAGCAACTCCCACTCTTCC
GAPDH R	TGGGTGGTCCAGGGTTTCTTACTCCTT
IL-1β F	AGTTGACGGACCCCAAA
IL-1β R	TCTTGTTGATGTGCTGCTG
IL-6 F	CAAAGCCAGAGTCCTTCAGAG
IL-6 R	AGCATTGGAAATTGGGGTAG

## Data Availability

The original contributions presented in this study are included in the article. Further inquiries can be directed to the corresponding author(s).

## Abbreviations

ALI: acute lung injury; ARDS: acute respiratory distress syndrome; BP: biological process; CC: cellular component; CETSA: cellular thermal shift assay; DEGs: differentially expressed genes; DEX: dexamethasone; GO: gene ontology; GSEA: gene set enrichment analysis; GSH: glutathione; H&E: hematoxylin and eosin; IL-1β: interleukin-1β; KEGG: Kyoto Encyclopedia of Genes and Genomes; LPS: lipopolysaccharide; MDA: malondialdehyde; MD: molecular dynamics; MF: molecular function; PCA: principal component analysis; ROS: reactive oxygen species; SOD: superoxide dismutase; SPR: surface plasmon resonance; TLR4: Toll-like receptor 4; TNF-α: tumor necrosis factor-α; WB: Western blot analysis.
